# Brefeldin A-Inhibited Guanine Nucleotide-Exchange Factor 1 (BIG1) Governs the Recruitment of Tumor Necrosis Factor Receptor-Associated Factor 2 (TRAF2) to Tumor Necrosis Factor Receptor 1 (TNFR1) Signaling Complexes

**DOI:** 10.3390/ijms17111869

**Published:** 2016-11-09

**Authors:** Takuya Noguchi, Mei Tsuchida, Yosuke Kogue, Christian Spadini, Yusuke Hirata, Atsushi Matsuzawa

**Affiliations:** 1 Department of Biochemistry, University of Lausanne, Chemin des Boveresses 155, CH-1066 Epalinges, Switzerland; christian.spadini88@gmail.com; 2 Laboratory of Health Chemistry, Graduate School of Pharmaceutical Sciences, Tohoku University, 6-3 Aoba, Aramaki, Aoba-ku, 980-8578 Sendai, Japan; mei.tsuchida.s3@dc.tohoku.ac.jp (M.T.); yosuke.kogue.t4@dc.tohoku.ac.jp (Y.K.); y-hirata@m.tohoku.ac.jp (Y.H.)

**Keywords:** tumor necrosis factor-α (TNF-α), apoptosis, JNK, TRAF2, BIG1

## Abstract

Tumor necrosis factor receptor-associated factor 2 (TRAF2) is a critical mediator of tumor necrosis factor-α (TNF-α) signaling. However, the regulatory mechanisms of TRAF2 are not fully understood. Here we show evidence that TRAF2 requires brefeldin A-inhibited guanine nucleotide-exchange factor 1 (BIG1) to be recruited into TNF receptor 1 (TNFR1) signaling complexes. In BIG1 knockdown cells, TNF-α-induced c-Jun N-terminal kinase (JNK) activation was attenuated and the sensitivity to TNF-α-induced apoptosis was increased. Since these trends correlated well with those of TRAF2 deficient cells as previously demonstrated, we tested whether BIG1 functions as an upstream regulator of TRAF2 in TNFR1 signaling. As expected, we found that knockdown of BIG1 suppressed TNF-α-dependent ubiquitination of TRAF2 that is required for JNK activation, and impaired the recruitment of TRAF2 to the TNFR1 signaling complex (complex I). Moreover, we found that the recruitment of TRAF2 to the death-inducing signaling complex termed complex II was also impaired in BIG1 knockdown cells. These results suggest that BIG1 is a key component of the machinery that drives TRAF2 to the signaling complexes formed after TNFR1 activation. Thus, our data demonstrate a novel and unexpected function of BIG1 that regulates TNFR1 signaling by targeting TRAF2.

## 1. Introduction

Adenosine diphosphate (ADP)-ribosylation factor (ARF) is a small guanosine triphosphate (GTP)-binding protein that plays a key role in the regulation of membrane trafficking and the organization of the cytoskeleton [1,2]. The activation state of ARFs is reciprocally regulated by ARF-GEFs (guanine nucleotide exchange factors) and GAPs (GTPase activating proteins) [3]. The ARF-GEFs have the Sec7 domain that catalyzes the exchange of guanine nucleotide on ARFs, and are divided into low- and high-molecular-weight ARF-GEFs [4]. Brefeldin A-inhibited guanine nucleotide-exchange factor 1 (BIG1) is classified as a high-molecular-weight ARF-GEF together with brefeldin A-inhibited guanine nucleotide-exchange factor 2 (BIG2) and Golgi-specific brefeldin A-resistance guanine nucleotide exchange factor 1 (GBF1), and these GEFs are also classified as brefeldin A (BFA)-sensitive ARF-GEFs [5,6,7]. BIG1 and BIG2 were originally isolated as a part of the macromolecular complex, and both preferentially activate ARF1 and ARF3 by catalyzing the replacement of ARF-bound guanosine diphosphate (GDP) with GTP to coordinate membrane trafficking between the Golgi apparatus and the plasma membrane [5,7,8,9]. The BIG-regulated membrane trafficking contributes to various cellular dynamics including cell spreading and adhesion, cell migration in wound healing, and neurite outgrowth [10,11,12]. The localization of BIG1 at the Golgi compartments has been observed in various culture cells, and the cyclic adenosine monophosphate (cAMP)-dependent protein kinase A (PKA) promotes nuclear accumulation of BIG1 [13]. On the other hand, it has been reported that BIG2 is associated with not only Golgi compartments but also recycling endosomes, and regulates tubulation of endosomal membranes [14]. Interestingly, BIG1 fails to compensate for the function of BIG2, indicating that the tubulation of endosomal membranes is regulated by BIG2-specific mechanisms [15]. Moreover, BIG1 and BIG2 are paired with different binding partners, whereas they interact with each other [4]. Thus, a series of recent studies of the BIGs strongly suggest that BIG1 and BIG2 have distinct and redundant functions.

Tumor necrosis factor-α (TNF-α) is a proinflammatory cytokine that induces a wide variety of cellular responses including cell growth, differentiation, immune responses, and cell death [16]. Two different receptors, named TNF receptor 1 (TNFR1) and TNF receptor 2 (TNFR2), can mediate signals from TNF-α. TNFR1 is ubiquitously expressed on almost all cells whereas only restricted cell types including neurons, endothelial cells, cardiac myocytes, thymocytes and mesenchymal cells express TNFR2. Engagement of TNFR1 by TNF-α rapidly leads to formation of a receptor complex referred to as the TNFR1 signaling complex (complex I), which elicits activation of the canonical nuclear factor-κB (NF-κB) pathways and the mitogen-activated protein (MAP) kinase pathways, such as the c-Jun N-terminal kinase (JNK) and p38 MAP kinase pathways, that preferentially induce anti-apoptotic and pro-inflammatory responses. Although the gene activation as a primary signaling outcome of TNFR1 activation contributes to cellular survival, TNFR1 can also induce cell death including apoptosis and necroptosis under certain circumstances. TNFR1-induced apoptosis is mediated by the cytosolic complex (complex II) formed as a result of rearrangement of complex I [17,18]. Complex II is a Fas-associated protein with death domain (FADD)-based complex that functions as a signaling hub to activate caspase-8. Meanwhile, further rearrangement of complex II occurs when caspase-8 activation is blocked, resulting in the formation of a necrosome that promotes activation of the necrotic pathways [19].

Tumor necrosis factor receptor-associated factor 2 (TRAF2), belonging to the TRAF family of proteins, is a really interesting new gene (RING) finger protein that participates in TNF-α-induced cellular responses as a signaling component of both complex I and complex II [20,21]. Upon binding of TNF-α to TNFR1, TRAF2 is recruited to TNFR1 through the interaction with TNFR-associated death domain protein (TRADD), and then recruits cellular inhibitor of apoptosis 1 and 2 (cIAP1/2) proteins to complex I. cIAP1/2 catalyzes the ubiquitination of receptor-interacting protein 1 (RIP1), which in turn elicits further recruitment of the essential components including inhibitor of nuclear factor kappa-B kinase (IKK) complex, the linear ubiquitin assembly complex (LUBAC) and transforming growth factor β-activated kinase 1 (TAK) 1 into complex I [16,22]. The contribution of TRAF2 to the downstream signaling has been investigated by the gene knockout studies in mice [23,24]. In particular, these studies revealed that TRAF2 plays critical roles in activation of the canonical NF-κB and the JNK pathways. However, since TRAF2-mediated NF-κB activation is compensated by tumor necrosis factor receptor-associated factor 5 (TRAF5), another member of TRAF family proteins, TRAF2 knockout cells exhibit only a mild reduction of the NF-κB activation [23]. Nevertheless, TRAF2 knockout cells exhibit increased sensitivity to TNF-α-induced apoptosis, suggesting that TRAF2 is required for NF-κB-independent mechanisms that protect against TNF-α-induced apoptosis. Similar effects of TRAF2 on cell death have been demonstrated in Fas or tumor necrosis factor-related apoptosis-inducing ligand (TRAIL) receptor signaling [25,26]. Moreover, a recent report has demonstrated that TRAF2 suppresses the induction of apoptosis through the mechanism of K48-linked polyubiquitination of activated caspase-8, which can account for the NF-κB-independent mechanism by which TRAF2 exerts anti-apoptotic activity [27].

Compelling evidence indicates that cellular responses mediated by TNFR1 are highly associated with the membrane trafficking of TNFR1 [17,28,29]. TNFR1 is internalized into signaling vesicles termed TNFR1 receptosomes after complex I formation, which is an essential process for ensuring proper signal transduction [29,30]. However, the involvement of the ARF-GEFs in TNFR1-medated cellular processes is largely unknown, except that it has been demonstrated that BIG2 regulates the constitutive release of TNFR1 exosome-like vesicles [31]. In this study, we found that BIG1 is involved in TNF-α signaling. Knockdown of BIG1 showed similar phenotypes of TRAF2 deficient cells that had decreased activation of JNK and increased apoptosis in response to TNF-α. We also found that BIG1 is required for the recruitment of TRAF2 to both complex I and complex II. Taken together, these results indicate that BIG1 regulates TNFR1 signaling by targeting TRAF2.

## 2. Results

### 2.1. BIG1 Is Involved in TNF-α-Induced JNK Activation

**Figure 1:**
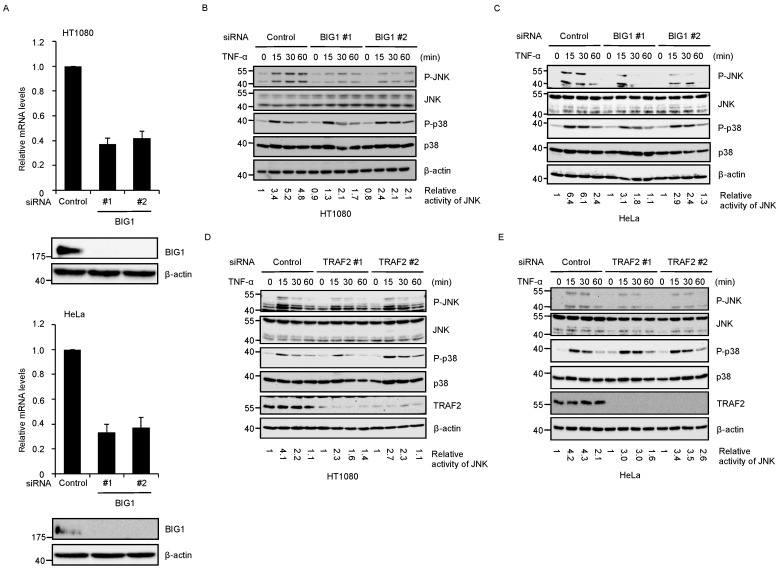
Brefeldin A-inhibited guanine nucleotide-exchange factor 1 (BIG1) is involved in tumor necrosis factor-α (TNF-α)-induced c-Jun N-terminal kinases (JNK) activation. (**A**) HT1080 or HeLa cells were transfected with small interfering RNA (siRNA) for negative control or BIG1 (BIG1 #1 or BIG1 #2). After 48 h, the relative mRNA levels of Big1 and protein expression of BIG1 were determined by quantitative RT-PCR and immunoblotting, respectively. Data shown are the mean ± standard error of the mean (SEM) (*n* = 3); (**B**–**E**) HT1080 (**B**) or HeLa cells (**C**) were transfected with siRNA for negative control or BIG1. HT1080 (**D**) or HeLa cells (**E**) were transfected with siRNA for negative control or tumor necrosis factor receptor-associated factor 2 (TRAF2). After 72 h, the cells were treated with 20 ng/mL TNF-α for the indicated periods. The cell extracts were subjected to immunoblotting with the indicated antibodies. The relative activity of JNK was quantified using ImageJ software.

In order to examine whether BIG1 is involved in TNFR1 signaling, we performed BIG1 knockdown experiments using HT1080 fibrosarcoma or HeLa cells that predominantly express TNFR1 rather than TNFR2 [32]. Both independent small interfering RNAs (siRNA) against BIG1 significantly reduced both mRNA and protein expression of BIG1 (Figure 1A). When BIG1 expression was knocked down by these siRNAs in HT1080 cells, immunoblotting analysis using the phospho-specific antibodies that can monitor the activation states revealed that TNF-α-induced JNK activation was attenuated in BIG1 knockdown cells, while that of p38 MAP kinase was unchanged (Figure 1B). Similar findings were observed in BIG1 knockdown HeLa cells (Figure 1C). Moreover, we found that TNF-α-induced JNK activation was also attenuated in TRAF2 knockdown cells as previous studies have demonstrated (Figure 1D,E) [23,24]. These results suggest that BIG1 is involved in TNF-α-induced JNK activation in a similar manner to TRAF2.

### 2.2. BIG1 Is Dispensable for TNF-α-Induced Degradation of IκBα

**Figure 2:**
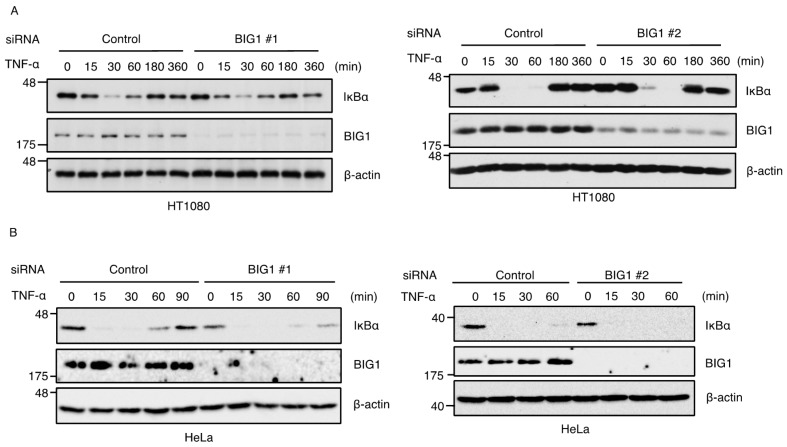

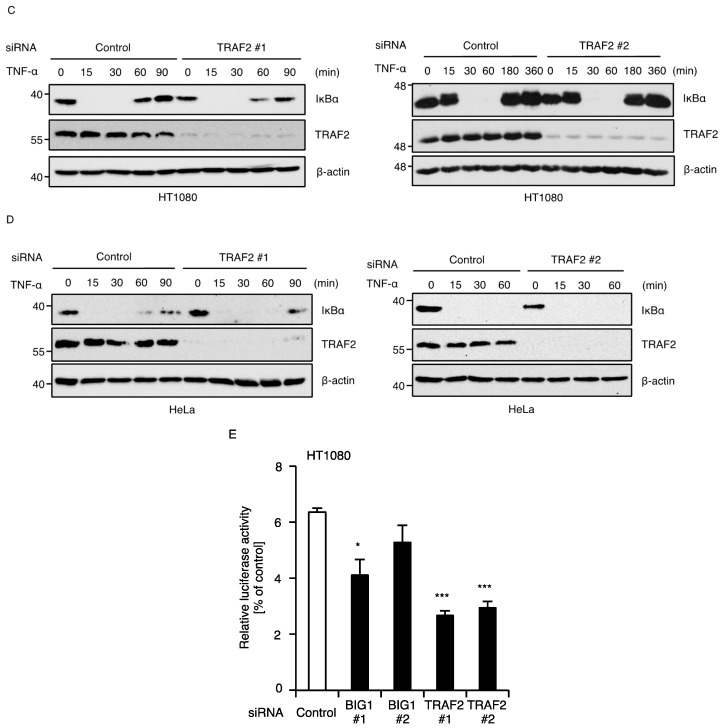
BIG1 is dispensable for TNF-α-induced degradation of IκBα. (**A**–**D**) HT1080 (**A**) or HeLa cells (**B**) were transfected with siRNA for negative control or BIG1. HT1080 (**C**) or HeLa cells (**D**) were transfected with siRNA for negative control or TRAF2. After 72 h, the cells were treated with 20 ng/mL TNF-α for the indicated periods. The cell extracts were subjected to immunoblotting with the indicated antibodies; (**E**) HT1080 were transfected with indicated siRNA. After 24 h, the cells were transfected with a plasmid mix containing a NF-κB luciferase reporter plasmid and a renilla luciferase plasmid for normalization together with indicated siRNA. After 48 h, cells were treated with 20 ng/mL TNF-α for 6 h. Firefly and renilla luciferase activities were quantified with dual luciferase reporter assay kit. Data shown are the mean ± SEM (*n* = 3). Statistical significance was tested using an unpaired Student’s *t*-test (vs. control cells); * *p* < 0.05, *** *p* < 0.001.

We next examined whether BIG1 knockdown affects the canonical NF-κB activation. In steady state condition, activation of the canonical NF-κB pathway is blocked by IκBα that inhibits NF-κB nuclear translocation, whereas, upon TNFR1 activation, IKK complex induces the phosphorylation-dependent degradation of IκBα, leading to the translocation and activation of NF-κB. As shown in Figure 2A, treatment with TNF-α promoted the degradation of IκBα, which occurred within 15 min and peaked at 30 min, and the expression levels of IκBα was returned to basal levels at 180 min, probably by the transcriptional activity of NF-κB. The kinetics of the degradation and the induction of IκBα were unaltered in both BIG1 knockdown HT1080 and HeLa cells (Figure 2A,B). Similar findings were observed in TRAF2 knockdown cells (Figure 2C,D). On the other hand, luciferase reporter assays of NF-κB displayed slightly reduced reporter activity in BIG1 knockdown cells when compared with control cells, although there was a statistically nonsignificant reduction (*p* = 0.16 vs. control) in the cells knocked down by BIG1 #2 siRNA (Figure 2E). These results suggest that BIG1 is dispensable for the degradation of IκBα, but it is possible that BIG1 is only partially involved in TNF-α-induced NF-κB activation through an alternative mechanism. Moreover, TRAF2 knockdown cells displayed a modest but significant reduction of the reporter activity without any effect on the degradation of IκBα, which is in agreement with data from previous studies using TRAF2-deficient cells (Figure 2C–E) [23,24]. Collectively, BIG1 knockdown cells displayed properties similar to those of TRAF2 in TNF-α-induced NF-κB activation, yet the extent of reduction in the reporter assay was limited in BIG1 knockdown cells.

### 2.3. Knockdown of BIG1 Increases Sensitivity to TNF-α-Induced Apoptosis

**Figure 3:**
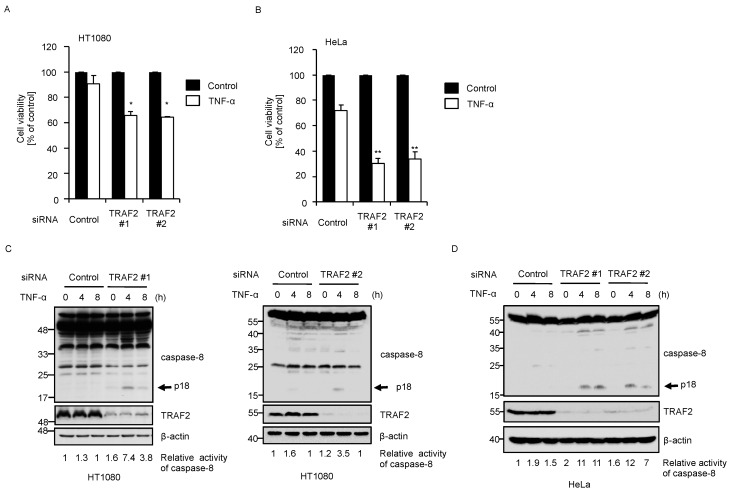
Knockdown of TRAF2 increases sensitivity to TNF-α-induced apoptosis. (**A**,**B**) HT1080 (**A**) or HeLa cells (**B**) were transfected with siRNA for negative control or TRAF2, and treated 72 h later with ± 50 ng/mL of TNF-α for 24 h. Cell viability was monitored with the phenazine methosulfate (PMS)/ 3-(4,5-dimethylthiazol-2-yl)-5-(3-carboxymethoxyphenyl)-2-(4-sulfophenyl)- 2H-tetrazolium, inner salt (MTS) assay, normalized to untreated control cells and expressed as mean ± SEM (*n* = 3). Statistical significance was tested using an unpaired Student’s *t*-test (vs. control cells); * *p* < 0.05, ** *p* < 0.01, (vs. control cells); (**C**,**D**) HT1080 (**C**) or HeLa cells (**D**) were transfected with siRNA for negative control or TRAF2. After 72 h, the cells were treated with 100 ng/mL TNF-α for the indicated periods. Cell extracts were subjected to immunoblotting with the indicated antibodies. The relative activity of caspase-8 was quantified using ImageJ software.

Since TNF-α responses in the early phase elicit the induction of anti-apoptotic proteins such as cellular FLICE-like inhibitory protein (c-FLIP), cIAP1/2 and TRAF2/5, the activation of cell death pathways is strongly suppressed [33,34]. However, the lack of the anti-apoptotic proteins usually allows caspase-8 activation and subsequent induction of apoptosis. For instance, TRAF2 knockout mouse embryonic fibroblasts (MEFs) exhibit increased sensitivity to TNF-α-induced apoptosis, and co-deletion of TRAF5 with TRAF2 further increases its sensitivity [23,24]. As shown in Figure 3A,B, TRAF2 knockdown reduced cell viability 24 h after the treatment with TNF-α. Moreover, in TNF-α-treated TRAF2 knockdown cells, we observed increased p18 fragments of caspase-8, which was a result of autocatalytic cleavage (auto-cleavage) of caspase-8 induced after the complex II formation (Figure 3C,D). These results indicate that knockdown of TRAF2 promotes TNF-α-induced apoptosis in both HT1080 and HeLa cells.

**Figure 4:**
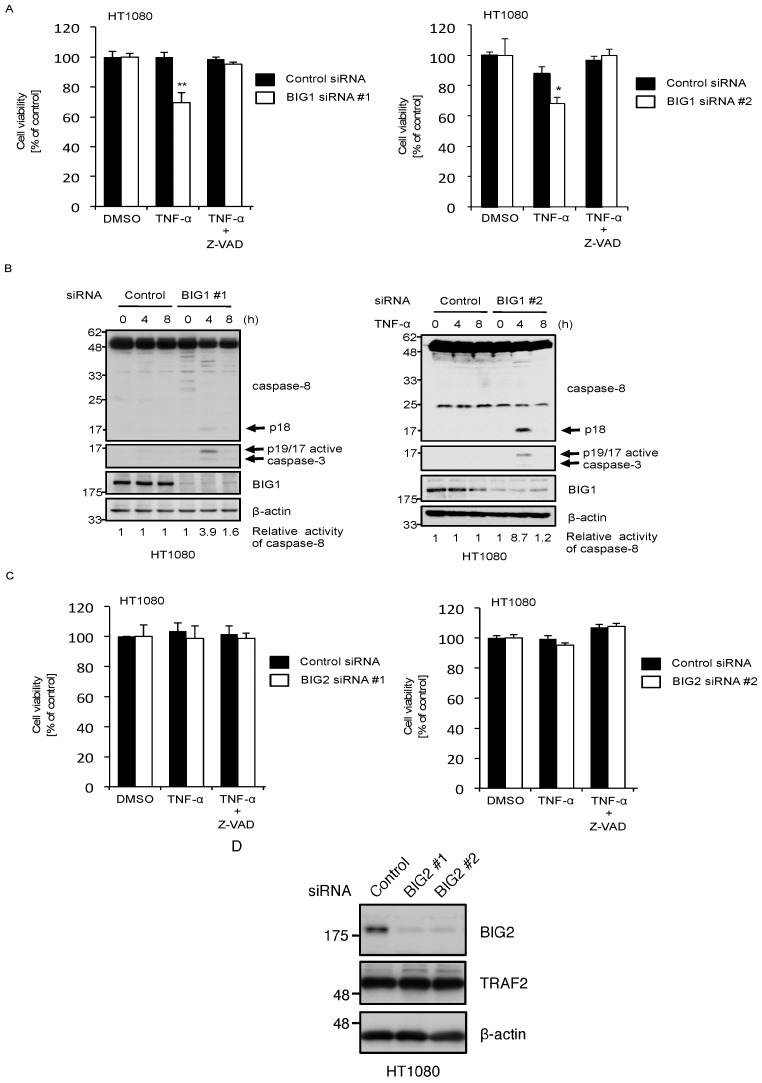
Knockdown of BIG1 increases sensitivity to TNF-α-induced apoptosis. (**A**) HT1080 cells were transfected with siRNA for negative control or BIG1, and treated 72 h later with ± 50 ng/mL of TNF-α for 24 h. DMSO (dimethy sulfoxide) was used as vehicle control. Cell viability was monitored with the PMS/MTS assay, normalized to untreated control cells and expressed as mean ± SEM (*n* = 3). Statistical significance was tested using an unpaired Student’s *t*-test (vs. control cells); * *p* < 0.05, ** *p* < 0.01, (vs. control cells); (**B**) HT1080 cells were transfected with siRNA for negative control or BIG1. After 72 h, the cells were treated with 100 ng/mL TNF-α for the indicated periods. Cell extracts were subjected to immunoblotting with the indicated antibodies. The relative activity of caspase-8 was quantified using ImageJ software; (**C**) HT1080 cells were transfected with siRNA for negative control or BIG2, and treated 72 h later ± 50 ng/mL of TNF-α for 24 h. DMSO was used as vehicle control. Cell viability was monitored with the PMS/MTS assay, normalized to untreated control cells and expressed as mean ± SEM (*n* = 3); (**D**) HT1080 cells were transfected with siRNA for negative control or BIG2. After 72 h, cell extracts were subjected to immunoblotting with the indicated antibodies.

We next examined whether knockdown of BIG1 affects the sensitivity to TNF-α-induced apoptosis. When siRNA-transfected cells were treated with TNF-α, we found that knockdown of BIG1 significantly reduced cell viability to a similar extent as TRAF2 knockdown cells (as shown in Figure 3A), and this was completely recovered when treated with a pan-caspase inhibitor Z-VAD-fmk (fluoromethylketone) (Figure 4A). Moreover, the cleavage of caspase-8 followed by caspase-3 activation was apparently accelerated in BIG1 knockdown cells (Figure 4B). On the other hand, knockdown of BIG2 did not have any effect on TNF-α-induced apoptosis, although BIG2 expression was sufficiently suppressed (Figure 4C,D). These results show that BIG1 is required for protecting cells from TNF-α-induced apoptosis, and BIG2 is not able to compensate its functions.

### 2.4. BIG1 Is Involved in the Recruitment of TRAF2 to the TNFR1 Signaling Complex

**Figure 5:**
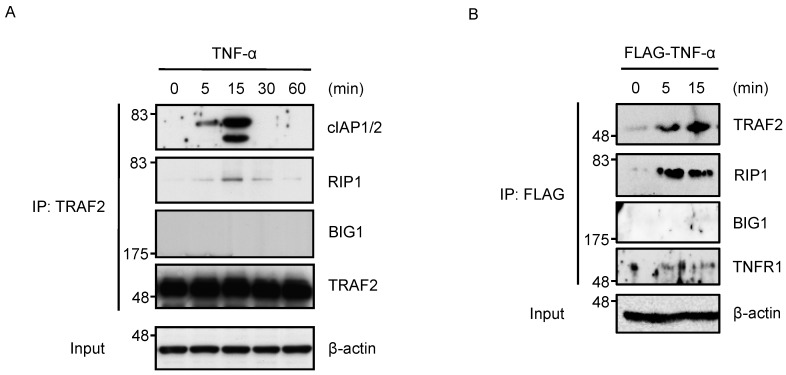

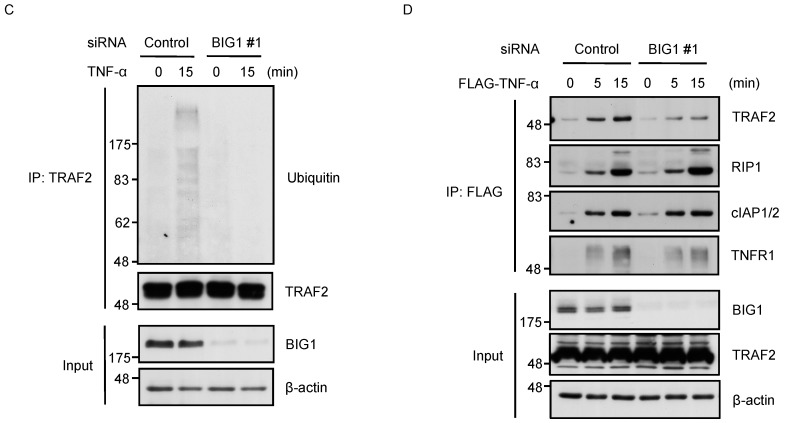
BIG1 is involved in the recruitment of TRAF2 to the TNF receptor 1 (TNFR1) signaling complex. (**A**,**B**) HT1080 cells were treated with 50 ng/mL TNF-α (A) or 2 μg/mL DYKDDDDK (FLAG)-TNF-α (B) for the indicated periods. The cell extracts were immunoprecipitated with anti-TRAF2 (**A**) or anti-FLAG (M2) (**B**) antibody followed by immunoblotting with the indicated antibodies; (**C**) HT1080 cells were transfected with siRNA for negative control or BIG1. After 72 h, the cells were treated with 50 ng/mL TNF-α for the indicated periods. The cells were lysed in lysis buffer supplemented with 10 mM N-ethylmaleimide (NEM), and subjected to immunoprecipitation with anti-TRAF2 antibody (1st-IP). The heat-denatured immunoprecipitates with lysis buffer containing 1% sodium dodecyl sulfate (SDS) to disrupt noncovalent protein-protein interactions were diluted 10 times with lysis buffer, and immunoprecipitated with anti-TRAF2 antibody (2nd-IP) followed by immunoblotting with the indicated antibodies; (**D**) HT1080 cells were transfected with siRNA for negative control or BIG1. After 72 h, the cells were treated with 2 μg/mL FLAG-TNF-α for the indicated periods. The cell extracts were immunoprecipitated with anti-FLAG (M2) antibody followed by immunoblotting with the indicated antibodies.

The signaling properties of TNF-α responses in BIG1 knockdown cells, which exhibited less activation of JNK and more activation of caspase-8, are similar to those in TRAF2 knockdown cells. These data prompted us to examine the functional relevance of BIG1 and TRAF2 in TNFR1 signaling. We first examined whether BIG1-TRAF2 interaction could be induced by TNF-α. When HT1080 cells treated with TNF-α, RIP1 and cIAP1 (well known as binding partners of TRAF2) were co-immunoprecipitated with TRAF2 peaked at 15 min after the treatment with TNF-α, whereas BIG1 was not evident in the co-immunoprecipitates of TRAF2 (Figure 5A). Moreover, when TNFR1 complex analysis using immunoprecipitation was performed, BIG1 was undetectable in the immunoprecipitates, even though TRAF2 was found in complex I (Figure 5B). These results suggest that the BIG1-TRAF2 interaction is either very weak or does not occur at all. Nevertheless, the responses of BIG1 knockdown cells to TNF-α yield suggestive evidence that functional defects of TRAF2 occur in BIG1 knockdown cells. We therefore examined whether BIG1 had effects on functional properties of TRAF2. In particular, TRAF2-mediated JNK activation induced by TNF-α requests E2 ubiquitin-conjugating enzyme (UBC) 13-dependent ubiquitination of TRAF2, which is observed shortly after TNF-α treatment [35]. In fact, we found that TRAF2 was ubiquitinated 15 min after the treatment with TNF-α (Figure 5C). Intriguingly, we also found that the ubiquitination of TRAF2 was strongly diminished in BIG1 knockdown cells when compared with control cells (Figure 5C). TRAF2 activity seems to be limited in BIG1 knockdown cells due to the impaired ubiquitination. In addition, considering that TRAF2 is recruited to complex I rapidly after TNF-α treatment, it is reasonable to assume that the immediate-type of TRAF2 ubiquitination occurs in complex I, where TRAF2 molecules are brought into close proximity enabling TRAF2 oligomerization and activation. We therefore speculate that BIG1 regulates TRAF2 recruitment to complex I. When complex I was analyzed by immunoprecipitation, we found that the recruitment of TRAF2 to complex I was impaired by BIG1 knockdown, while that of RIP1 and cIAP1 occurred to a similar extent as in control cells (Figure 5D). These results show that BIG1 is involved in the recruitment of TRAF2 to complex I.

### 2.5. BIG1 Is Involved in the Recruitment of TRAF2 to the Death-Inducing Complex

**Figure 6:**
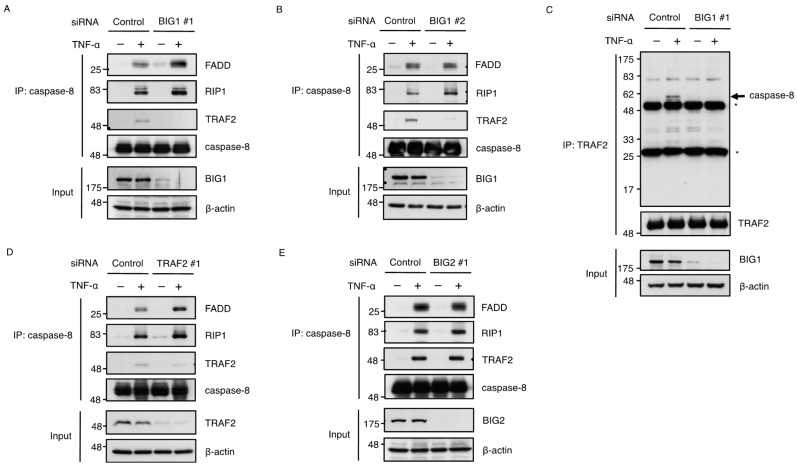
BIG1 is involved in the recruitment of TRAF2 to the death-inducing complex. (**A**–**E**) HT1080 cells were transfected with indicated siRNAs. After 72 h, the cells were treated with 50 ng/mL TNF-α for 4 h. The cell extracts were immunoprecipitated with caspase-8 or TRAF2 antibody followed by immunoblotting with the indicated antibodies. Asterisks (*) in (**C**) indicate the heavy and light chains of IgG.

Given that BIG1 regulates the TRAF2 recruitment to the complex I, TRAF2 may fail to assemble into the death-inducing complex (complex II) in BIG1 knockdown cells. To explore this possibility, we analyzed the complex II formation using caspase-8 antibody. To promote formation of the complex II, HT1080 cells were treated with TNF-α in combination with the protein-synthesis inhibitor cycloheximide (CHX) which blocks NF-κB-dependent induction of anti-apoptotic proteins that interfere with complex II formation. As shown in Figure 6A, in co-treatment with TNF-α and CHX, we found that the components of the complex II such as FADD, RIP1 and TRAF2 were co-immunoprecipitated with caspase-8 in control cells, meaning that typical complex II was certainly assembled. On the other hand, we found that knockdown of BIG1 inhibited only the recruitment of TRAF2 into complex II (Figure 6A,B). Furthermore, when subjected to immunoprecipitation with TRAF2 antibody, we also found that caspase-8, which co-immunoprecipitated with TRAF2, was severely impaired in BIG1 knockdown cells, suggesting that BIG1 is required for the recruitment of TRAF2 to complex II (Figure 6C). Otherwise, we observed that the RIP1 recruitment to the complex II in BIG1 knockdown cells was slightly increased with a reciprocal decrease in TRAF2 recruitment (Figure 6A,B). Interestingly, a similar trend was observed in TRAF2 knockdown cells (Figure 6D). These data raise the possibility that TRAF2 limits the access of RIP1 to complex II, and that the lack of TRAF2 allows potentiated RIP1 recruitment and subsequent caspase-8 activation, which is similar to the cIAP deficiency as previously reported [36]. Thus, enhanced RIP1 recruitment to complex II of BIG1- or TRAF2-knockdown cells may contribute to increased susceptibility to apoptosis. On the contrary, no obvious differences were detected in the amount of FADD, RIP1 and TRAF2 recruitment to complex II of cells treated with control or BIG2 siRNA (Figure 6E). These results, taken together, suggest that BIG1 regulates the recruitment of TRAF2 to the complex II, which cannot be compensated by BIG2.

## 3. Discussion

In the present study, we provide evidence that BIG1, one of the high-molecular-weight ARF-GEFs, participates in TNFR1 signaling through the mechanism governing TRAF2 recruitment to the TNFR1 signaling complexes. In BIG1 knockdown cells, TNF-α-induced JNK activation was attenuated whereas the degradation of IκBα was unaltered. Moreover, knockdown of BIG1 promoted TNF-α-induced caspase-8 and subsequent caspase-3 activation, leading to cell apoptosis. These signaling properties observed in BIG1 knockdown cells were similar to those in TRAF2 knockdown cells, which alluded to the possibility that BIG1 is an upstream regulator of TRAF2. Indeed, we found that BIG1 knockdown abrogated TNF-α-induced TRAF2 ubiquitination. Moreover, we also found that BIG1 knockdown impaired the recruitment of TRAF2 to both complex I and complex II. Collectively, these findings suggest that BIG1 upregulates the activities of TRAF2 by governing the TRAF2 recruitment to TNFR1 signaling complexes. On the other hand, BIG2 knockdown failed to promote TNF-α-induced apoptosis as shown in Figure 4C. Moreover, BIG2 knockdown did not affect the recruitment of TRAF2 to complex II (Figure 6E). These findings indicate that BIG2 is not involved in the anti-apoptotic mechanisms of TRAF2 in TNFR1 signaling. Although BIG1 and BIG2 share a similar structure, accumulating evidence suggests that they have distinct subcellular localizations, functions, and binding partners [4]. The action of BIG1 on TRAF2-mediated functions in TNFR1 signaling appears to be a distinct and redundant role of BIG1. Although the precise mechanisms by which BIG1 drives TRAF2 to the complexes remain unknown, BIG1 may selectively determine the behaviors of TRAF2 through the direct or indirect mechanisms. Considering that we could not detect the BIG1-TRAF2 interaction and the recruitment of BIG1 to the TNFR1 complexes (as shown in Figure 5A,B), BIG1 appears not to serve as an adaptor protein for TRAF2. Although the direct interaction of BIG1 with TRAF2 could not be completely eliminated, TRAF2 is more likely to be regulated by the indirect mechanisms mediating inputs from BIG1. In this regard, it has been reported that TRAF2 stably associated with the membrane-organizing protein caveolin-1 at subcellular compartments of enrichment subjacent to the plasma membrane, and TRAF2-induced JNK activation is required for the translocation of TRAF2 to the insoluble membrane rafts where TRAF2 recruits the upstream kinases of JNK [35,37]. These previous findings implicate BIG1 in the trafficking mechanisms that are pivotal to appropriate activation of TRAF2. To evaluate the trafficking mechanisms mediated by BIG1, testing whether the GEF activity of BIG1 is required for the trafficking of TRAF2 is one of the most important issues. However, in this study, the reconstitution experiments using catalytically inactive mutants of BIG1 to determine the requirement of the BIG1-GEF activity did not work due to extremely low expression levels of the BIG1 transgenes. For the same reason, analysis of the functional interaction between BIG1 and TRAF2 was not enough to exclude the possibility that BIG1 functions as an adaptor protein for TRAF2. Thus, although further studies are needed to elucidate the BIG-1-mediated mechanisms, TRAF2-mediated functions in TNFR1 signaling may be dominated by the trafficking mechanisms, in which BIG1 may play a key role.

TNFR1 favors both survival and death signaling pathways. Its signaling diversity seems to be exquisitely determined by intracellular trafficking that controls the correct intracellular distribution of TNFR1. At the plasma membrane, TNFR1 forms complex I, which principally activates the survival signaling pathways. In turn, the complex I changes from a plasma membrane complex to the receptosome as a signaling vesicle after its internalization, which is essential for the late-phase process including apoptosis and necroptosis [17,29,38]. Thus, elucidation of the trafficking mechanisms associated with the internalization of TNFR1 and the formation of TNFR1 receptosome is crucial for a comprehensive understanding of TNF-α-induced cell death. At the beginning of this study, we evaluated the contribution of BIG1 to the trafficking mechanisms of TNFR1. However, TNF-α-dependent interaction of caspase-8 and FADD, which is an index of complex II formation, was evident even in the absence of BIG1, indicating that BIG1 is dispensable for the internalization of TNFR1 and the formation of TNFR1 receptosomes (Figure 6A,B). Nonetheless, we found that BIG1 participates in the TNFR1 signaling by targeting TRAF2. Our findings provide insight into the mechanisms by which the intracellular trafficking commits to the proper disposition of not only TNFR1 but also each component of TNFR1 complexes. Dysregulation of the trafficking mechanisms may contribute to the pathogenesis of various diseases including cancer. Uncovering the unidentified trafficking mechanisms in the TNFR1 signaling is therefore the key to understanding the molecular basis and developing novel therapeutic strategies for TNF-α-related diseases.

## 4. Materials and Methods

### 4.1. Cell Culture and Reagents

HT1080 and HeLa cells were grown in Dulbecco’s Modified Eagle Medium (DMEM), 10% heat-inactivated fetal bovine serum (FBS), and 1% penicillin-streptomycin solution, at 37 °C under a 5% CO_2_ atmosphere. FLAG-TNF-α was produced and purified as described [39,40]. TNF-α was purchased from Enzo Life Sciences. siRNAs were purchased from Qiagen (Big1 #1: SI04254593, BIG1#2: SI04168892, BIG2#1: SI04323305, BIG2#2: SI04292554, TRAF2 #1: SI00129619, TRAF2 #2: SI03073455). All Stars negative control siRNA (Qiagen, Hilden, Germany) was used as a control. siRNAs were transfected using Lipofectamine RNAiMAX (Invitrogen, Carlsbad, CA, USA), according to the manufacturer’s protocol. Antibodies used in this study are listed in Table A1.

### 4.2. Immunoblot

Cells were lysed in lysis buffer (1% Nonidet P-40, 50 mM Tris pH 7.4, 150 mM NaCl, 5 mM ethylenediaminetetraacetic acid (EDTA)), containing one cOmplete Protease Inhibitor Cocktail tablet (Roche, Basel, Switzerland) per 50 mL. Cell extracts were resolved by SDS-PAGE, transferred to nitrocellulose or polyvinylidene difluoride (PVDF) membranes, blocked with phosphate buffered saline, 0.5% Tween 20, 5% powdered skim milk, probed with the indicated antibodies and revealed using the ECL reagent (GE Healthcare, Little Chalfont, UK).

### 4.3. NF-κB Reporter Assay

NF-κB reporter assays were performed essentially as described [41]. Cells were transfected using Lipofectamine 2000 (Invitrogen) with a plasmid mix containing a NF-κB luciferase reporter plasmid, a renilla luciferase plasmid for normalization, and an empty plasmid. After 48 h, cells were treated with TNF-α for 6 h. Firefly and renilla luciferase activities were quantified with dual luciferase reporter assay system (Promega, Madison, WI, USA).

### 4.4. Colorimetric Cell Viability Assay

At 60 h post-transfection, siRNA-transfected cells were plated in 96-well plates at 20–30,000 cells per well in 100 μL of medium. Then, 12 h later, cells were treated with TNF-α in 100 μL of medium for about 16 h. Following this, 20 μL of a 1:20 (*v*/*v*) mixture of 0.9 mg/mL PMS (phenazine methosulfate) and 2 mg/mL MTS (3-(4,5-dimethylthiazol-2-yl)-5-(3-carboxymethoxyphenyl)-2-(4-sulfophenyl)-2*H*-tetrazolium), (Promega, Madison, WI, USA) solution was added into each well. Following color development for 2–4 h, absorbance was measured at 492 nm.

### 4.5. TNFR1 Complex Analysis by Immunoprecipitation

The complex I formation initiated upon TNF-α stimulation was analyzed by use of FLAG-TNF-α. HT1080 cells were stimulated for the indicated times with 2 μg FLAG-TNF-α and lysed in 1 mL lysis buffer (20 mM Tris-HCl pH 7.4, 150 mM NaCl, 0.2% Nonidet P40, 10% glycerol, and complete protease inhibitor cocktail) for 15 min on ice. Lysates were immunoprecipitated with 20 μL anti-FLAG M2 magnetic beads for 4 h at 4 °C. Beads were recovered by the magnet and washed five times with 1 mL of lysis buffer before analysis by SDS-PAGE and immunoblotting. To analyse complex II, HT1080 cells were lysed in lysis buffer. After centrifugation, supernatants were precleared with 20 µL of Sepharose 6B (Sigma St. Louis, MO, USA) for 1 h at 4 °C, and then immunoprecipitated with caspase-8 antibody using protein G-Sepharose (GE Healthcare, Little Chalfont, UK). Beads were extensively washed with lysis buffer before immunoblot analysis.

## 5. Conclusions

In this study, we found that BIG1 knockdown cells exhibited similar properties to those of TRAF2 in TNFR1-mediated cellular responses. These data thus prompted us to examine whether BIG1 functions an upstream regulator of TRAF2 in TNFR1 signaling. Indeed, knockdown of BIG1 suppressed TNF-α-dependent ubiquitination of TRAF2, and impaired the recruitment of TRAF2 to the TNFR1 signaling complexes. These results suggest that BIG1 is required for the recruitment of TRAF2 to the signaling complexes formed after TNFR1 activation. Altogether, our data demonstrate a novel function of BIG1 that regulates TNFR1 signaling by targeting TRAF2.

## Appendix A

**Table A1:** Antibodies used in this study are listed.

Antibody	Species, Name (Source)
β-actin	rabbit pAb, ab8337 (Abcam or Santa Cruz)
BIG1	rabbit pAb, H-200 (Santa Cruz)
BIG2	rabbit pAb (Bethyl Laboratories)
caspase-3 cleaved	rabbit pAb (Cell Signaling)
caspase-8 for IP	goat pAb, p20 C-20 (Santa Cruz)
caspase-8 for IB	mouse mAb, Clone 12F5 (Enzo Life Sciences)
cIAP1	rat mAb, 1E1-1-10 (Enzo Life Sciences)
FADD	mouse mAb (Becton and Dickinson)
FLAG	mouse mAb, M2 (Sigma)
IκBα	rabbit pAb (Cell signaling)
p38, phospho	rabbit pAb (Cell signaling)
JNK, phospho	rabbit pAb (Biosource or Cell signaling)
RIP1	mouse mAb (Becton and Dickinson)
TRAF2	mouse mAb (Becton and Dickinson or Santa Cruz)
Ubiquitin	mouse mAb, P4D1 (Santa Cruz)
